# Efficacy and indications of gamma knife radiosurgery for recurrent low-and high-grade glioma

**DOI:** 10.1186/s12885-023-11772-8

**Published:** 2024-01-05

**Authors:** Ying Sun, Peiru Liu, Zixi Wang, Haibo Zhang, Ying Xu, Shenghui Hu, Ying Yan

**Affiliations:** 1 Department of Radiation Oncology, General Hospital of Northern Theater Command, 110016 Shenyang, China; 2 Beifang Hospital of China Medical University, 110016 Shenyang, China; 3 Graduate School of Dalian Medical University, 116000 Dalian, China

**Keywords:** Recurrent glioma, Gamma knife radiosurgery, Prognosis, Indication

## Abstract

**Purpose:**

To investigate the indications and efficacy of gamma knife radiosurgery (GKRS) as a salvage treatment for recurrent low-and high-grade glioma.

**Methods:**

This retrospective study of 107 patients with recurrent glioma treated with GKRS between 2009 and 2022, including 68 high-grade glioma (HGG) and 39 low-grade glioma (LGG) cases. The Kaplan-Meier method was used to calculate the overall survival (OS) and progression-free survival (PFS). The log-rank test was used to analyze the multivariate prognosis of the Cox proportional hazards model. Adverse reactions were evaluated according to the *Common Terminology Criteria for Adverse Events version 4.03*. The prognostic value of main clinical features was estimated, including histopathology, Karnofsky performance status (KPS), recurrence time interval, target location, two or more GKRS, surgery for recurrence, site of recurrence, left or right side of the brain and so on.

**Results:**

The median follow-up time was 74.5 months. The median OS and PFS were 17.0 months and 5.5 months for all patients. The median OS and PFS were 11.0 months and 5.0 months for HGG, respectively. The median OS and PFS were 49.0 months and 12.0 months for LGG, respectively. Multivariate analysis showed that two or more GKRS, left or right side of the brain and brainstem significantly affected PFS. Meanwhile, the KPS index, two or more GKRS, pathological grade, and brainstem significantly affected OS. Stratified analysis showed that surgery for recurrence significantly affected OS and PFS for LGG. KPS significantly affected OS and PFS for HGG. No serious adverse events were noted post-GKRS.

**Conclusion:**

GKRS is a safe and effective salvage treatment for recurrent glioma. Moreover, it can be applied after multiple recurrences with tolerable adverse effects.

## Introduction

Glioma is the most common malignant cancer of the central nervous system, and its treatment is one of the most challenging problems in neuro-oncology [1–4]. Its high morbidity and lethality rates seriously threaten patients’ lives [5]. Currently, there is no standard treatment plan for recurrent glioma in China, and the available treatment options include re-surgery, gene targeting therapy, tumor electric field therapy, chemotherapy, and re-irradiation [6–10]. Re-surgery is not suitable for all patients, and the risk of surgery and recurrence rate is relatively high due to the rich blood flow and infiltrative growth of glioma. Systemic chemotherapy can be administered with regimens such as carmustine, temozolomide (TMZ), or PCV (prednisone, carmustine, vincristine) [11], but usually provides only minimal long-term benefit.

GKRS has recently become an increasingly popular treatment option for clinicians because of its short treatment period, low economic burden, and few adverse effects. However, several studies have been performed to apply GKRS to the salvage treatment of patients with recurrent glioma, but the results remain to be considered [12–13]. A study from the Netherlands evaluated the efficacy of GKRS in the treatment of patients with recurrent glioblastoma and demonstrated a local control rate of 27% for high-grade gliomas and 50% for LGG, with median progression-free survival and overall survival (PFS and OS) of 10.5 months and 34.4 months, respectively, for all patients [14]. Dodoo et al. [15] found that patients with grade IV gliomas who underwent GKRS had a median survival of up to 11.3 months, with a two-year survival rate of 22.9%. Sharma et al. investigated the role of GKRS in patients with rGBM, with an estimated median OS after salvage SRS of 11.0 months (95% CI 7.1–12.2), median PFS of 4.4 months (95% CI 3.7-5.0), and total tumor volume, KPS score, and homogeneity index (i.e., more heterogeneous plans), more heterogeneous plans were independent predictors of OS [12]. Because gliomas often grow infiltratively and are not clearly demarcated from surrounding tissues, and because the GKRS dose is concentrated and the marginal dose attenuates more rapidly, the treatment of glioblastoma with GKRS is still controversial. This article will provide a detailed analysis of the treatment experience over the past 13 years.

## Methods

### Patient selection and baseline clinical characteristics

In this retrospective study, patients with recurrent gliomas who underwent Head Gamma Knife at the Radiotherapy Department of the General Hospital of the Northern Theater of Operations (GHNTO) from September 2009 to December 2022 were searched through the Head Gamma Knife Database of the Radiotherapy Department and Electronic Medical Record System (EMRS) software, follow-up visits by phone every three months. Patients were followed up regularly after treatment in accordance with clinical guidelines, and imaging data were obtained from our hospital database. Inclusion criteria are as follows: (1) patients whose initial treatment plan included a definitive pathological diagnosis of glioma (WHO grade I-IV) by surgery or puncture biopsy. (2) Confirmed primary tumor recurrence (image confirmed or pathologically confirmed by repeat surgery or puncture biopsy). (3) Treatment options after recurrence mainly include Gamma Knife radiosurgery, and the detailed treatment plan can be obtained by searching the Gamma Knife database in the Department of Radiotherapy. (4) The patient’s general condition (gender, age, KPS score, etc.), detailed treatment plan (pathology at the first consultation, adjuvant treatment modality after the first consultation, age at recurrence, surgery after recurrence, pathology after recurrence, KPS score at the time of recurrence of Gamma Knife treatment, etc.), post-treatment side effects, disease progression, etc. can be retrieved through the EMRS medical record system, or follow-up visits can be conducted by telephone and outpatient clinic. The exclusion criteria are (1) Insufficient clinical data. (2) Inability or unwillingness to cooperate with the study program and follow-up. (3) Individual characteristics that may lead to serious adverse reactions or even death after Gamma Knife treatment. (4) Comorbidities such as congestive heart failure, cirrhosis, other tumors, acute infections, etc. that may affect survival or efficacy. (5) Lack of autonomy or inability to attend the 3-month follow-up visit. (6) Loss of patient visits.

We gathered the clinical characteristics of patients including demographics (sex, age), pre-GKRS treatment specifics (initial surgical time, multiple craniotomies, surgery-to-GKRS interval, adjuvant treatment after initial surgery, KPS scale), and GKRS treatment parameters (number of targets, volume of targets, maximum dose, marginal dose, whether multiple GKRS, concurrent/adjuvant chemotherapy) through clinical notes, radiology reports, demographic data, and telephone follow-up. For patients treated with multiple targets in the same GKRS regimen, we focused on the parameters of total lesion volume for the first GKRS treatment. The general status of the patients was assessed according to the KPS scale [16]. Pre- and post-GKRS clinical characteristics are summarized in Table 1.

**Table 1:** Baseline clinical characteristics of all Patients pre- or post-GKRS [%]

Patient characteristics	Number of patients (%)	Patient characteristics	Number of patients (%)
**Sex**		**KPS**	
Female	58(54.2)	≥80	48(44.9)
Male	49(65.8)	<80	59(55.1)
**Pathological grade**		**Number of targets**	
LGG	39(36.4)	Single	45(42.1)
HGG	68(63.6)	Multiple	62(57.9)
**Adjuvant treatment after initial surgery**		**Total volume of targets**	
		≥18.5 cm^3^	51(47.7)
Chemoradiotherapy	51(47.7)	<18.5 cm^3^	49(52.3)
EBRT only	25(23.4)	**Maximum dose**	
TMZ only	5(4.7)	≥24.5 Gy	54(50.5)
GKRS only	10(9.3)	<24.5 Gy	53(49.5)
None	16(15.0)	**Marginal dose**	
**Age at GKRS procedure**		≥12.5 Gy	54(50.5)
≥50 years	58 (54.2)	<12.5 Gy	53(49.5)
<50 years	49(45.8)	**Two and more GKRS**	
**Recurrence time interval**		Yes	36(33.6)
≥15 months	54(50.5)	No	71(66.4)
<15 months	53(49.5)	**Bevacizumab used after recurrence**	
**Surgery for recurrence**		Yes	14(13.1)
Yes	27(25.2)	No	93(86.9)
No	80(74.8)		

### GKRS procedure

**Fig. 1:**
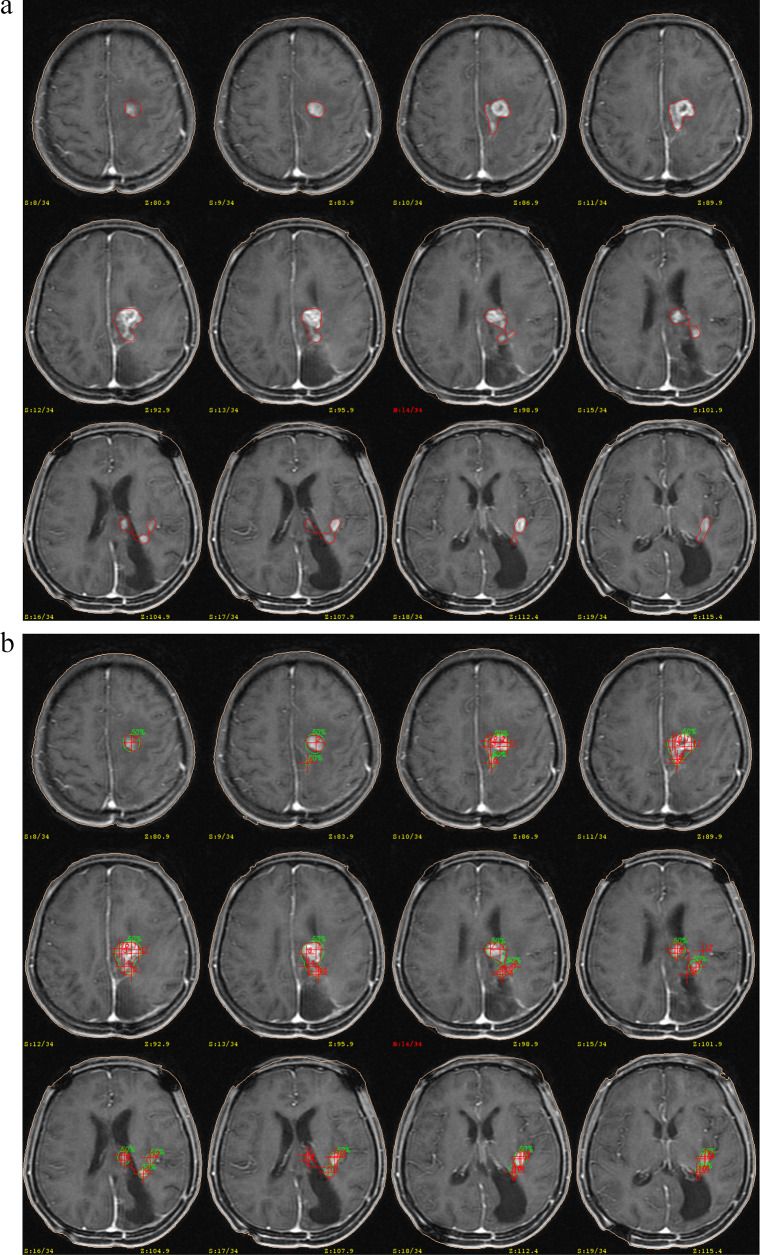
Distribution of marginal dose (**a**) and maximum dose (**b**) by treatment volume

**Fig. 2:**
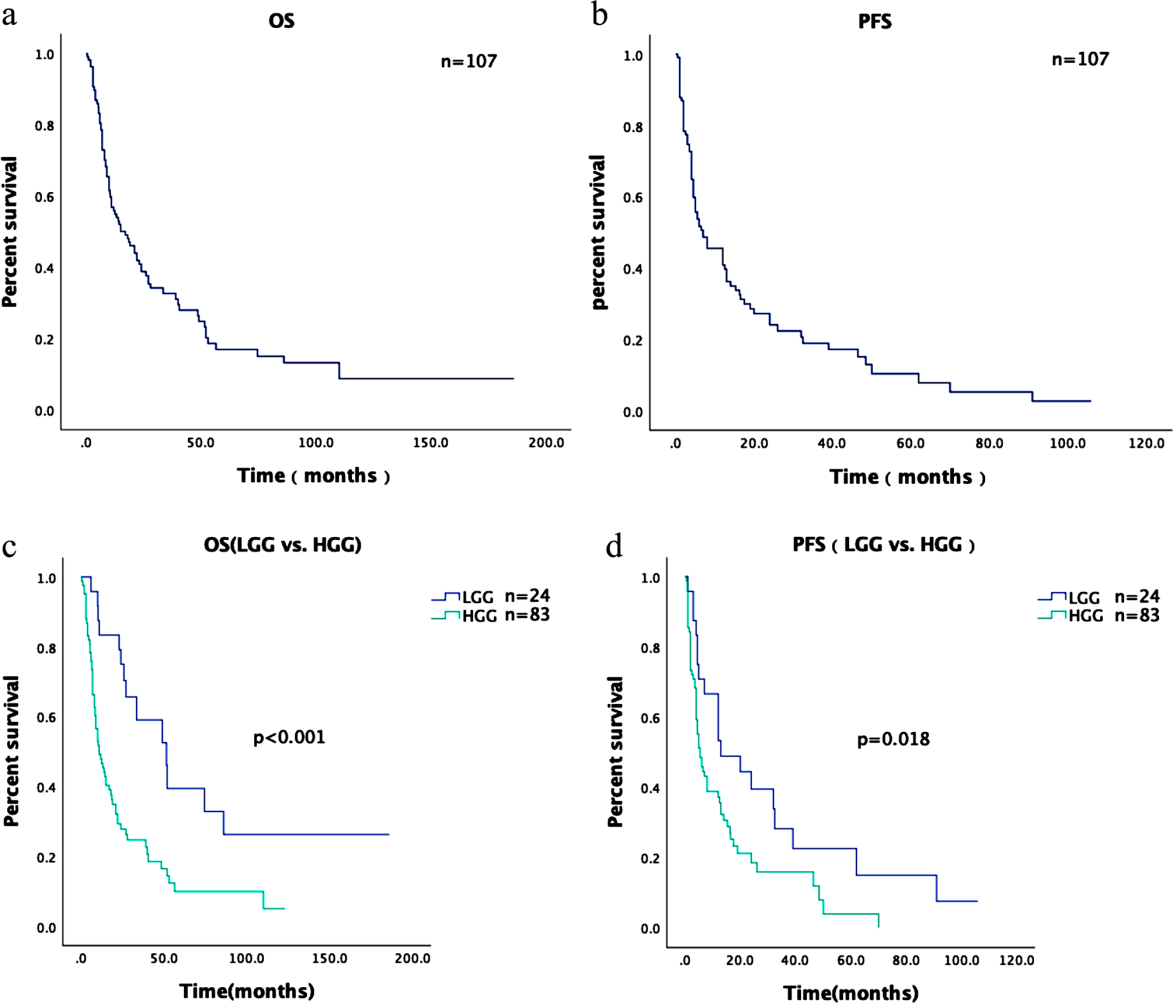
Kaplan-Meier survival after GKRS for (**a**) OS of all patients, (**b**) PFS of all patients, (**c**) Comparison of OS of LGG and HGG, (**d**) Comparison of PFS of LGG and HGG

According to the magnetic resonance imaging (MRI) data before treatment, patients were fitted with a stereotactic head frame under local anaesthesia. They underwent a 3.0T MRI examination with intravenous gadolinium contrast after that. The 3.0T MRI machine produced by GE company was used to obtain the positioning image, which was transmitted to the gamma treatment planning system through a particular network. Stereotactic radiosurgery and dose planning were then performed in consultation with a neurosurgeon, radiation oncologist, and medical physicist. The target was defined as the contrast-enhancing lesion on the T1 weighted axial images that were obtained with a slice thickness of 3.0 mm (Fig. 1). Target delineation was limited to the target-enhancing lesion only for progressive LGG and HGG, as confirmed by the American Society for Radiation Oncology (ASTRO) guidelines [17]. The median target volume was 18.5 cm^3^ (1.6-124.1 cm^3^), the median maximum dose was 25 Gy (13.5–42 Gy), and the median marginal dose was 13.0 Gy (7.5–24.5 Gy). We showed the relationship of the treatment volume compared with its marginal dose (Fig. 2a) and estimated maximal dose (Fig. 2b) in Fig. 2. The lesions that were larger than 3.5 cm in diameter or located in critical functional areas (such as the brain stem) were treated twice after several weeks.

### Drug treatment after GKRS

The patients were given a mannitol injection of 125 mL BID and a dexamethasone injection of 10 mL once daily intravenously to reduce and prevent the occurrence of brain edema. Forty-four patients received TMZ periodic chemotherapy (150–200 mg/m^2^) after GKRS, 1–5 days, 28 days as a cycle, and 21 patients received dose density scheme (75–100 mg/m^2^), 1–21 days, 28 days as a cycle, or 100–150 mg/m^2^, 1–7 days, 14 days as a cycle. One patient was treated with PCV; this was not analyzed because of the statistical insignificance of the small number. Fourteen patients were treated with 7.5 mg/kg of bevacizumab every 3 weeks; 5 cases were treated with bevacizumab combined with TMZ.

The OS was the time from the beginning of GKRS to death or the last follow-up time, and PFS was the time from the end of GKRS to local tumor progression or the last follow-up. The follow-up time was defined as the time from GKRS to death or the last follow-up time. Treatment‑related toxicities were scored using the *Common Terminology Criteria for Adverse Events version 4.03*.

### Statistical analysis

Data are presented as median with the range. The Pearson correlation coefficient was obtained through linear regression analysis. Survival analysis was done by the log-rank test on Kaplan-Meier survival estimates. Multivariate prognostic analysis was performed using the Cox proportional hazards model. Statistics were calculated with SPSS 26.0 software, values with *p* < 0.05 were considered statistically significant.

## Results

### Patient characteristics

We analyzed the characteristics of 107 patients (Table 2), including 68 patients with HGG and 39 patients with LGG, and the median age of all patients was 50.5 (17–80) years. All patients had histologically confirmed glioma. 105 patients had undergone at least one operation before GKRS and 2 patients were diagnosed by histopathological biopsy (both HGG). 8 patients (2 cases of LGG and 6 cases of HGG) had no definite time for disease progression after GKRS but a specific time of death. 73 patients had received adjuvant treatment after the first surgery, including chemoradiotherapy (47.7%), EBRT (23.4%), TMZ (5%), GKRS (9.3%), and the other 16 patients (15%) had none. 36 patients had received two or more GKRS after the recurrence. Univariate analysis demonstrated that prognostic factors associated with OS and PFS were primary pathological grade, recurrence time interval, surgery for recurrence, KPS, number of targets, two or more GKRS, brainstem, and encephalocele recurrence; central dose and peripheral dose were correlated with PFS.

**Table 2:** Univariate analysis of OS and PFS in all patients pre- and post-GKRS

Characteristics	Number of patients (%)	Median PFS (95% CI) *p*-value	Median OS (95% CI) *p*-value
**Sex**		0.458	0.425
Female	58(54.2)	7.0(1.4-12.6)	21.0(8.5-33.5)
Male	49(65.8)	6.0(2.6-9.4)	14.0(9.8-18.1)
**Pathological grade**		0.018	0.000
LGG	39(36.4)	13.0(1.1-24.9)	49.0(22.7-75.3)
HGG	68(63.6)	5.5(3.4-7.6)	11.0(6.6-15.4)
**Adjuvant treatment after first surgery**		0.808	0.877
Chemoradiotherapy	51(47.7)	6.5(3.9-9.1)	11.0(5.3-16.7)
EBRT	25(23.4)	12.0(1.4-22.6)	17.0(0.7-33.3)
TMZ	5(4.7)	5.5(4.4-6.6)	18.0(11.6-24.4)
GKRS	10(9.3)	8.0(0.0-22.7)	33.5(18.1-48.8)
None	16(15.0)	5.0(0.0-24.6)	12.5(3.3-21.7)
**Age at GKRS procedure**		0.938	0.804
≥50 years	58 (54.2)	8.0(5.2-10.8)	18.0(11.1-24.9)
<50 years	49(45.8)	6.5(0.1-12.9)	13.2(4.0-22.4)
**Recurrence time interval**		0.034	0.012
≥15 months	54(50.5)	12.0(6.1-17.9)	23.0(8.7-37.2)
<15 months	53(49.5)	5.0(3.5-6.7)	14.0(8.0-20.0)
**Surgery for recurrence**		0.013	0.002
Yes	27(25.2)	17.5(0.0-43.8)	52.0(2.9-101.1)
No	80(74.8)	5.5(3.6-7.4)	14.0(7.7-20.8)
**KPS**		0.004	0.000
≥80	48(44.9)	14.0(6.1-21.9)	40.0(16.1-63.9)
<80	59(55.1)	4.5(3.7-5.3)	10.0(7.9-12.1)
**Number of targets**		0.130	0.008
Single	45(42.1)	12.0(4.8-19.2)	24.0(3.7-44.3)
Multiple	62(57.9)	5.0(2.2-7.8)	11.0(6.6-15.4)
**Total volume of target**		0.624	0.544
≥18.5 cm^3^	51(47.7)	7.0(4.0-10.0)	14.3(5.7-22.9)
<18.5 cm^3^	49(52.3)	7.0(0.7-13.3)	21.0(9.7-32.3)
**Maximum dose**		0.255	0.122
≥24.5 Gy	53(49.5)	13.0(6.9-19.1)	22.0(15.0-29.0)
<24.5 Gy	54(50.5)	4.5(3.5-6.5)	12.0(7.7-16.3)
**Marginal dose**		0.300	0.165
≥12.5 Gy	54(50.5)	12.0(5.9-18.1)	22.0(14.0-30.0)
<12.5 Gy	53(49.5)	5.0(2.7-7.3)	12.0(8.2-16.7)
**Two or more GKRS**		0.031	0.001
Yes	36(33.6)	13.0(9.0-17.0)	33.5(1.8-65.2)
No	71(66.4)	5.0(2.9-7.1)	10.5(7.7-13.3)
**Target location**		0.000	0.000
In or adjacent field	63(58.9)	13.0(8.1-18.0)	26.0(18.9-33.1)
Out of field	44(41.1)	4.0(2.3-5.7)	8.0(4.8-11.2)
**Site of recurrence**			
		0.889	0.629
Frontal	52(48.6)	7.0(4.2-9.8)	21.0(14.4-27.6)
Others	55(51.4)	8.0(0.0-17.5)	11.0(6.1-15.9)
		0.496	0.299
Temporal	40(37.4)	8.0(0.0-18.0)	9.0(2.9-15.1)
Others	67(62.6)	6.5(4.0-9.0)	21.0(13.5-28.5)
		0.416	0.687
Occipital	10(9.3)	14.0(0.0-33.3)	11.0(7.2-14.8)
Others	97(90.7)	6.5(3.8-9.2)	18.0(10.9-25.1)
		0.804	0.390
Parietal	15(14.1)	12.5(3.0-22.0)	18.5(0.0-48.2)
Others	92(85.9)	7.0(4.0-10.0)	17.0(9.8-24.2)
		0.000	0.000
Brainstem	3(2.8)	1.5(0.7-2.3)	3.0(1.4-4.6)
Others	104(97.2)	8.0(3.2-12.8)	18.0(11.1-24.9)
		0.032	0.000
Encephalocoele	14(13.1)	2.0(0.0-5.5)	8.0(4.6-11.4)
Others	93(86.9)	8.0(2.9-13.1)	21.0(13.2-28.8)
**Left or right side of the brain**		0.001	0.007
Left	40(37.4)	7.0(0.0-17.9)	14.3(7.0-21.0)
Right	44(41.1)	12.0(6.4-17.6)	39.0(15.0-63.0)
Bilateral	23(21.5)	4.5(1.6-7.4)	10.5(7.3-13.6)
**Bevacizumab used after recurrence**		0.360	0.633
Yes	14(13.1)	8.0(2.7-13.3)	10.0(0.0-23.9)
No	93(86.9)	7.0(2.9-11.0)	18.0(9.4-26.6)

*Abbreviations: LGG* Low-grade glioma, *HGG* High-grade glioma, *WHO* World health organization, *EBRT* External beam radiotherapy, *TMZ* Temozolomide, *GKRS* Gamma knife radiosurgery, *KPS* Karnofsky performance status, *Target location* In relation to the previously irradiated tumor volume

After receiving gamma knife radiosurgery, 107 patients were monitored for local progression rate at 3, 6, 9, and 12 months. The rates were 71.96%, 47.66%, 39.25%, and 32.71%, respectively. The local progression rate at 3, 6, 9, and 12 months in the 24 patients with LGG were 87.50%, 70.83%, 66.67% and 50.00%. The remaining 83 patients with HGG and their local progression rate were 67.47%, 40.96%, 31.33%, and 27.71% at 3, 6, 9, and 12 months, respectively. The survival analysis and prognostic factors by PFS and OS are shown in Table 2, and the Kaplan-Meier survival curves are shown in Fig. 2(a–d). The median follow-up time was 79.5 months (95% CI:60.6–98.4). By the end of the study, 65 patients died during the follow-up period. All patients’ median OS and PFS were 12.5 months (95% CI:7.8–17.1) and 5.0 months (95% CI:3.8–6.2), respectively. The median OS and PFS were 10.0 months (95% CI:4.9–15.1) and 4.5 months (3.0–6.0) for HGG, respectively. The median OS and PFS were 27.0 months (95% CI:0.0–56.0) and 12.0 months (95% CI: 3.9–20.1) for LGG, respectively.

Multivariate analysis showed that the Left or right side of the brain, two or more GKRS and the brainstem were independent influencing factors for PFS (*p = 0.018, p = 0.023, p* = 0.041, *p* = 0.034, respectively). KPS index, pathological grade, two or more GKRS, and brainstem were independent risk factors for the OS (*p* = 0.001, *p* = 0.031, *p* = 0.002, *p* = 0.042 respectively) (Table 3).

**Table 3:** Cox proportional-hazards multivariate models of PFS and OS in all patients

Parameters	OS		PFS	
	HR (95% CI)	*p-*value	HR (95% CI)	*p-*value
KPS	0.411(0.242-0.698)	0.001	0.593(0.344-1.021)	0.059
Pathological grade	0.481(0.247-0.936)	0.031	0.780(0.405-1.503)	0.458
left or right side of the brain(left)	0.804(0.430-1.500)	0.492	0.415(0.200-0.859)	0.018
left or right side of the brain(right)	0.495(0.260-0.944)	0.033	0.452(0.227-0.898)	0.023
Two or more GKRS	2.680(1.431-5.017)	0.002	2.483(1.405-4.388)	0.041
Brainstem recurrence	0.248(0.065-0.952)	0.042	0.254(0.072-0.900)	0.034

Stratified analysis showed that surgery for recurrence was an independent influencing factor of OS for LGG (*p* = 0.009). Site of recurrence (encephalocele) and surgery for recurrence were the influencing factors of PFS for LGG (*p* = 0.009, *p* = 0.023) (Table 4). KPS > 80 and taking two or more GKRS were the influencing factors of OS for HGG (*p* = 0.001, *p* = 0.011). KPS > 80 and having a recurrence at the site of the brainstem were the influencing factors of PFS for HGG (*p* = 0.046, *p* = 0.018) (Table 5).

**Table 4:** Cox proportional-hazards multivariate models of PFS and OS in LGG patients

Parameters	OS		PFS	
	HR (95% CI)	p-value	HR (95% CI)	p-value
Encephalocoele recurrence	0.439(0.089-2.166)	0.312	0.071(0.010-0.516)	0.009
Surgery for recurrence	8.682(1.715-43.944)	0.009	4.951(1.245-19.696)	0.023

**Table 5:** Cox proportional-hazards multivariate models of PFS and OS of HGG patients

Parameters	OS		PFS	
	HR (95% CI)	p-value	HR (95% CI)	p-value
KPS	0.374(0.209-0.668)	0.001	0.571(0.330-0.990)	0.046
Two or more GKRS	2.194(1.196-4.042)	0.011	0.561(0.247-1.247)	0.167
Brainstem recurrence	0.261(0.067-1.009)	0.052	0.220(0.063-0.767)	0.018

After conducting further subgroup analysis, it was found that the KPS and target location were the influencing factors of PFS for patients with grade 3 gliomas (*p* ≡ 0.006,*p* ≡ 0.009). Additionally, KPS was also identified as a independent influencing factors of OS for patients with grade 3 gliomas (*p* ≡ 0.041) (Table 6). Furthermore, Site of recurrence (brainstem) was found to be an independent prognostic factor of OS and PFS for patients with grade 4 glioblastoma (*p* = 0.008, *p* = 0.047) (Table 7).

**Table 6:** Cox proportional-hazards multivariate models of PFS and OS of grade 3 patients

Parameters	OS		PFS	
	HR (95% CI)	p-value	HR (95% CI)	p-value
KPS	0.280(0.112-0.699)	0.006	0.571(0.330-0.990)	0.041
Target location	0.267(0.099-0.722)	0.009	0.459(0.177-1.185)	0.107

**Table 7:** Cox proportional-hazards multivariate models of PFS and OS of grade 4 glioma patients

Parameters	OS		PFS	
	**HR (95% CI)**	**p-value**	**HR (95% CI)**	**p-value**
Brainstem recurrence	0.022(0.001-0.370)	0.008	0.100(0.010-0.974)	0.047

### Adverse reactions

74 patients (84%) had no adverse reactions, 9 patients (10.2%) had a mild headache (grade I-II), and 4 patients (4.5%) had mild dizziness, which was relieved after symptomatic treatment with mannitol and hormone. Nausea occurred in 3 patients (3.4%), and nausea accompanied by vomiting in 1 (1.1%), which was relieved after antiemetic and brain dehydration treatment. 2 patients with fatigue and weakness were relieved after rest. 1 patient had mild lethargy, which was relieved after three days. No severe adverse reactions occurred. The specific adverse reactions are shown in Table 8.

**Table 8:** Adverse events after GKRS

Adverse reactions	Number of LGG patients (%)	Average duration	Number of HGG patients (%)	Average duration
None	28(87.5)	–	46(82.1)	–
Headache (grade I)	2(6.3)	2d	4(7.1)	3d
Headache (grade II)	1(3.1)	3d	2(3.6)	5d
Dizziness (grade I)	1(3.1)	1d	3(5.4)	2d
Nausea and vomiting	1(3.1)	2d	2(3.6)	2d
Fatigue (grade I)	0(0)	–	2(3.6)	3d
Somnolence (grade I)	0(0)	–	1(1.8)	3d
Excessive fatigue	0(0)	–	–	–

## Discussion

Glioma is the most common tumors of the central nervous system in adults. They originate in the brain’s neuroglia and are characterized by high aggressiveness and metastasis. According to the 2021 WHO classification of central nervous system tumors [18], gliomas are classified as grades 1 to 4, with grades 1 and 2 being LGG and grades 3 and 4 being HGG. Several studies [19–20] have significantly advanced the understanding of glioma pathogenesis to improve the prognosis of glioma patients.

LGG is more suitable for reoperation because of its poor radiosensitivity. However, the exceptional location makes some patients more ideal for GKRS. HGG is highly invasive and tends to recur. The survival time of patients with recurrent glioblastoma is limited [21–23]. The risk of adverse reoperation or radiotherapy events may increase, and they tend to accept GKRS. Larson Da [24] et al. found that the factors influencing the survival time of all grades of glioma after radiotherapy were young age, high KPS index, small tumor volume, and single lesion. However, in our study, young age and single lesion had no significant relationship with PFS and OS. Still, the high KPS index significantly improved PFS and OS in patients, especially a KPS score greater than 80. Our follow-up found that the main factors affecting the KPS index were postoperative status, including whether there was neurological dysfunction, limb movement disorder, epilepsy, fatigue, etc. Further stratified analysis showed that the KPS index was the influencing factor of OS in patients with HGG. KPS was an independent influencing factor of PFS in patients with HGG.

The results of this study indicate that the number of GKRS treatments and the location of the recurrence （brainstem） are independent factors that affect the OS and PFS of patients with glioma. Further analysis showed that these factors had a more significant impact on patients with HGG, suggesting that those with recurrent HGG who had fewer GKRS treatments and a recurrence in the brainstem were more likely to have a poor prognosis.

In comparison to LGG patients, those with HGG have a less favorable prognosis and limited survival. As a result, we directed our focus towards HGG patients. Further, we refined their classification into “grade 3 glioma” and “grade 4 glioblastoma” to enhance the accuracy of Gamma Knife efficacy predictions for patients at different stages. Our subgroup analysis revealed that the KPS index is an independent prognostic factor for HGG and a robust prognostic indicator for patients with stage 3 gliomas. This implies that the KPS score of patients with grade 3 gliomas is more indicative of their prognosis than those with grade 4 glioblastomas. Patients with grade 3 gliomas and high KPS scores can anticipate a more favorable prognosis and longer survival. Patients diagnosed with grade 4 glioblastoma in HGG have a poor prognosis, especially if brainstem metastases occur. The survival rate of such patients becomes precarious. It is recommended that patients with grade 3 gliomas focus on rehabilitating and maintaining their physical and functional status. On the other hand, patients with grade 4 glioblastomas should follow their doctor’s instructions for three-monthly follow-ups and seek early intervention if a new lesion emerges, particularly in a rare location such as the brainstem.

Experts recommend early secondary surgery for recurrent glioma [25]. This study confirms that reoperation after recurrence is an independent prognostic factor affecting LGG OS and PFS. Patients who underwent secondary surgery after recurrence had a much longer median survival than those who did not undergo secondary surgery.

Although early secondary surgery is prognostically favorable, endogenous brainstem or encephalocele metastases are considered unfeasible for microsurgery due to their specific location, and stereotactic radiosurgery is deemed to be a recommended approach for the treatment of brainstem metastases, and it should be considered especially for patients with good physical status [26–27]. This study suggested that PFS was worse in patients with glioma when there was a recurrence in a specific location (brainstem, encephalocele). At the same time, the effect on OS is not significant—considering that some patients presenting with recurrent foci in such areas are more prone to glioma recurrence and shorter PFS when surgery or the accurate dose of Gamma knife is not possible due to the specificity of the location.

We also report the location of the recurrent focus and its relationship to the primary focus significantly affects the survival of patients on the left or right side of the brain. Left Bilateral had a significant association with PFS. Scoccianti et al. [28] found that it was suitable to choose stereotactic radiosurgery (12–15 Gy/F) when the target volume is smaller than 12.5 mL for patients with recurrent glioma, the median OS was 7.5–16 months and the median PFS was 4.6-7.0 months. Several studies have shown that GKRS may be more suitable for patients with small, focal, or nodular recurrent glioma [29–32]. One study found that the OS after SRS was significantly better for patients with a target volume smaller than 14 cm^3^ [32]. GKRS treatment for large lesions may increase the incidence of side effects. For patients with large single lesions, we would make the GKRS two-fractional irradiation, which not only increases the cumulative tumor dose but also reduces the dose to normal tissues and decreases the incidence of side effects.

**Fig. 3:**
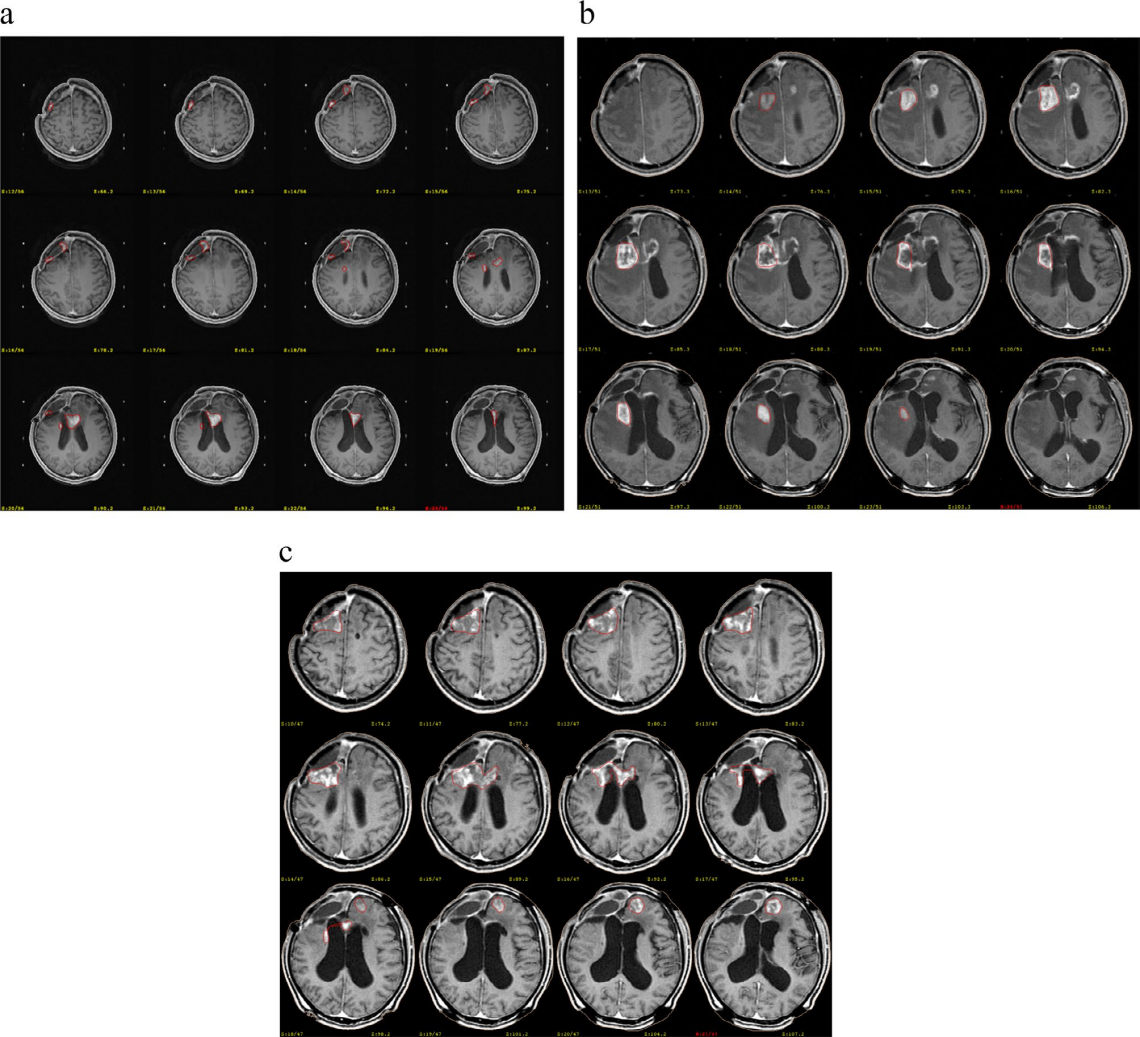
Illustrative case of a 54-year-old man who had 3 times GKRS treatments with rGBM (WHO III) following standard concurrent chemoradiotherapy and 13 cycles of adjunct chemotherapy. He exhibited an excellent response to salvage GKRS for each time of the tumor recurrence, which was on Nov 30, 2020 (**a**), Sep 9, 2021 (**b**) and Feb 28, 2023 (**c**). The right color lines of this figure are the target location. By the time of follow-up, the patient’s condition was stable and did not progress again

This study’s univariate and multivariate analysis showed that repeated GKRS significantly prolonged the OS with HGG and improved the patients’ life quality (Fig. 3). This is consistent with the findings of Sadik ZHA et al. GKRS can be safely used as a salvage treatment for recurrent glioma and may improve survival rates in HGG patients with minimal burden. Multiple GKRS treatments may benefit HGG patients in terms of OS [14]. However, our study did not find a significant effect of multiple GKRS treatments on PFS. Nonetheless, a survey conducted by Cheon et al. reported [33–34] that multiple GKRS treatments led to longer PFS only if the patient is recurrent with good activity status scores and limited tumor volume.

GKRS has apparent advantages in treating central nervous system tumors, shown by accurate localization and high-dose focused irradiation of the focal area. In contrast, the normal tissues around the tumor are better protected, which can reduce the radioactive brain damage to normal brain tissue. In contrast, patients with recurrent glioma often have already received radiotherapy, and the optic chiasm, optic nerve, brainstem and normal brain tissue may have reached maximum tolerance before GKRS treatment. External radiation therapy with EBRT is usually limited for recurrent glioma, and the dose is not too high. This study shows that the maximum dose in the target area and the marginal dose are prognostic factors for PFS. Therefore, PFS in these patients is directly affected by the adequacy of the dose in the target area. In the present study, 84% of patients with recurrent glioma had no adverse effects and grade III-IV serious adverse effects. Thus, compared with conventional radiotherapy, GKRS of recurrent glioma can increase the target dose and minimize the risk of side effects.

Although stereotactic radiosurgery is a feasible treatment, systemic therapy should be addressed. Some studies have shown that patients with recurrent glioblastoma receiving bevacizumab after GKRS treatment have a median survival time of 18 months [35]. The one-year survival rate after SRS is 73%, and the grade III toxicity is lower (9%). Compared with the patients without bevacizumab, they have higher survival benefits (12 vs. 18 months). Gutin PH et al. found that adding bevacizumab also increased OS in a hypo-fractionated stereotactic regimen [36]. However, some studies reported that it has rarely been significantly associated with improved OS in multivariable models [37]. We also did not find it had a significant correlation with the PFS and OS, and it may be due to the long follow-up period in this study and the number of patients treated with bevacizumab was small. However, from the clinical performance of patients with bevacizumab, the symptoms of brain edema were significantly relieved after medication, and the quality of life was improved. Next, we will collect more cases to prove the application of bevacizumab after GKRS may be a valuable adjuvant treatment.

### Study limitation

The limitations of this paper are as follows: First, since retrospective studies rely on historical data that have been collected, these data may be incomplete, inaccurate or inconsistent. Data quality issues may lead to biased or misleading results. During data collection, we try to use reliable data sources and ensure the accuracy and completeness of the data. Incomplete or inaccurate data are appropriately processed and analyzed. Second, retrospective studies may be at risk of selection bias, i.e., bias in the selection of the study sample. This bias may be due to subjectivity in sample selection, incomplete records, or lack of representativeness. Selection bias may lead to inaccurate and biased study results. During sample selection, we try to select a representative sample and avoid selection bias. Some non-representative treatment pathologies were excluded to ensure diversity and representativeness of the sample. The sample size of this study needs to be enlarged. This paper included 107 clinical cases of recurrent glioma patients who performed GKRS regimens in our hospital recent years, and increasing the sample size would further improve the persuasive power of clinical evidence. Third, retrospective studies may be at risk of confounding bias, i.e., the presence of other potential influences related to the study factors (age、tumor location、pathology grade) that may have an impact on the study results. Confounding bias may lead to inaccurate and misleading study results. We use multivariate analysis methods or other statistical methods to deal with the effects of confounders. This paper classified patients with LGG and HGG concerning the malignant degree typing of glioma. Adopting a classification model including cytomorphology and molecular pathology in the future will provide a more accurate and comprehensive assessment of the therapeutic effect of GKRS.

## Conclusion

GKRS, as a salvage treatment for recurrent glioma, is safe and effective. GKRS can significantly prolong the OS and PFS of patients, improve their quality of life, and prolong their survival period, especially for patients with small recurrent lesions, short intervals, high KPS, and who received multiple GKRS treatments.

## Data Availability

The datasets used and analyzed during the current study are available from the corresponding author upon reasonable request.

## List of abbreviations

GKRS: Gamma knife radiosurgery
HGG: Ligh-grade glioma
LGG: Low-grade glioma
Gy: Gray
OS: Overall survival
PFS: Progression-free survival
KPS: Karnofsky performance status
TMZ: Temozolomide
PCV: Prednisone, carmustine, vincristine
CI: Confidence interval
MRI: Magnetic resonance imaging
ASTRO: American society for radiation oncology
SRS: Stereotactic radiosurgery

## References

[1] Xu S, Tang L, Li X, Fan F, Liu Z (2020). Immunotherapy for glioma: current management and future application. Cancer Lett.

[2] Gladson CL, Prayson RA, Liu WM (2010). The pathobiology of glioma tumors. Annu Rev Pathol.

[3] Bi J, Chowdhry S, Wu S, Zhang W, Masui K, Mischel PS (2020). Altered cellular metabolism in gliomas - an emerging landscape of actionable co-dependency targets. Nat Rev Cancer.

[4] Reifenberger G, Wirsching HG, Knobbe-Thomsen CB, Weller M (2017). Advances in the molecular genetics of gliomas - implications for classification and therapy. Nat Rev Clin Oncol.

[5] Morgan LL (2015). The epidemiology of glioma in adults: a state of the science review. Neuro Oncol.

[6] She L, Su L, Liu C (2022). Bevacizumab combined with re-irradiation in recurrent glioblastoma. Front Oncol.

[7] Szklener K, Bilski M, Nieoczym K, Mańdziuk D, Mańdziuk S (2023). Enhancing glioblastoma treatment through the integration of tumor-treating fields. Front Oncol.

[8] Kazmi F, Soon YY, Leong YH, Koh WY, Vellayappan B (2019). Re-irradiation for recurrent glioblastoma (GBM): a systematic review and meta-analysis. J Neurooncol.

[9] Dono A, Mitra S, Shah M, Takayasu T, Zhu JJ, Tandon N, Patel CB, Esquenazi Y, Ballester LY (2021). PTEN mutations predict benefit from Tumor treating fields (TTFields) therapy in patients with recurrent glioblastoma. J Neurooncol.

[10] Khan KI, Ramesh P, Kanagalingam S, Zargham Ul Haq F, Victory Srinivasan N, Khan AI, Mashat GD, Hazique M, Khan S (2022). Bevacizumab-Induced Hypertension as a potential physiological clinical biomarker for improved outcomes in patients with recurrent Glioblastoma Multiforme: a systematic review. Cureus.

[11] Leone A, Colamaria A, Fochi NP, Sacco M, Landriscina M, Parbonetti G, de Notaris M, Coppola G, De Santis E, Giordano G et al. Recurrent Glioblastoma treatment: state of the art and future perspectives in the Precision Medicine Era. Biomedicines 2022, 10(8). 10.3390/biomedicines10081927 PMC9405902 36009473

[12] Larson EW, Peterson HE, Lamoreaux WT, MacKay AR, Fairbanks RK, Call JA, Carlson JD, Ling BC, Demakas JJ, Cooke BS (2014). Clinical outcomes following salvage Gamma Knife radiosurgery for recurrent glioblastoma. World J Clin Oncol.

[13] Crowley RW, Pouratian N, Sheehan JP (2006). Gamma knife Surgery for Glioblastoma Multiforme. Neurosurg Focus.

[14] Sadik ZHA, Hanssens PEJ, Verheul JB, Beute GN, Te Lie S, Leenstra S, Ardon H (2018). Gamma knife radiosurgery for recurrent gliomas. J Neurooncol.

[15] Dodoo E, Huffmann B, Peredo I, Grinaker H, Sinclair G, Machinis T, Enger PO, Skeie BS, Pedersen PH, Ohlsson M (2014). Increased survival using delayed gamma knife radiosurgery for recurrent high-grade glioma: a feasibility study. World Neurosurg.

[16] Karnofsky DA, Burchenal JH, Macleod CM (1949). The clinical evaluation of chemotherapeutic agents in cancer. Evaluation of Chemotherapeutic agents.

[17] Cabrera AR, Kirkpatrick JP, Fiveash JB, Shih HA, Koay EJ, Lutz S, Petit J, Chao ST, Brown PD, Vogelbaum M (2016). Radiation therapy for glioblastoma: executive summary of an American Society for Radiation Oncology evidence-based clinical practice Guideline. Pract Radiat Oncol.

[18] Louis DN, Perry A, Wesseling P, Brat DJ, Cree IA, Figarella-Branger D, Hawkins C, Ng HK, Pfister SM, Reifenberger G (2021). The 2021 WHO classification of tumors of the Central Nervous System: a summary. Neuro Oncol.

[19] Server A, Kulle B, Gadmar ØB, Josefsen R, Kumar T, Nakstad PH (2011). Measurements of diagnostic examination performance using quantitative apparent diffusion coefficient and proton MR spectroscopic imaging in the preoperative evaluation of Tumor grade in cerebral gliomas. Eur J Radiol.

[20] Nabors LB, Portnow J, Ahluwalia M, Baehring J, Brem H, Brem S, Butowski N, Campian JL, Clark SW, Fabiano AJ (2020). Central Nervous System Cancers, Version 3.2020, NCCN Clinical Practice guidelines in Oncology. J Natl Compr Canc Netw.

[21] Biswas T, Okunieff P, Schell MC, Smudzin T, Pilcher WH, Bakos RS, Vates GE, Walter KA, Wensel A, Korones DN (2009). Stereotactic radiosurgery for glioblastoma: retrospective analysis. Radiat Oncol.

[22] Lamborn KR, Yung WKA, Chang SM, Wen PY, Cloughesy TF, DeAngelis LM, Robins HI, Lieberman FS, Fine HA, Fink KL (2008). Progression-free survival: an important end point in evaluating therapy for recurrent high-grade gliomas. Neurooncology.

[23] Wong ET, Hess KR, Gleason MJ, Jaeckle KA, Kyritsis AP, Prados MD, Levin VA, Yung WK (1999). Outcomes and prognostic factors in recurrent glioma patients enrolled onto phase II clinical trials. J Clin Oncol.

[24] Larson DA, Gutin PH, McDermott M, Lamborn K, Sneed PK, Wara WM, Flickinger JC, Kondziolka D, Lunsford LD, Hudgins WR (1996). Gamma knife for glioma: selection factors and survival. Int J Radiat Oncol Biol Phys.

[25] Capelle L, Fontaine D, Mandonnet E, Taillandier L, Golmard JL, Bauchet L, Pallud J, Peruzzi P, Baron MH, Kujas M (2013). Spontaneous and therapeutic prognostic factors in adult hemispheric World Health Organization Grade II gliomas: a series of 1097 cases: clinical article. J Neurosurg.

[26] Sinclair G, Benmakhlouf H, Martin H, Maeurer M, Dodoo E (2019). Adaptive hypofractionated gamma knife radiosurgery in the acute management of brainstem metastases. Surg Neurol Int.

[27] Jung EW, Rakowski JT, Delly F, Jagannathan J, Konski AA, Guthikonda M, Kim H, Mittal S (2013). Gamma Knife radiosurgery in the management of brainstem metastases. Clin Neurol Neurosurg.

[28] Scoccianti S, Francolini G, Carta GA, Greto D, Detti B, Simontacchi G, Visani L, Baki M, Poggesi L, Bonomo P (2018). Re-irradiation as salvage treatment in recurrent glioblastoma: a comprehensive literature review to provide practical answers to frequently asked questions. Crit Rev Oncol Hematol.

[29] Mahajan A, McCutcheon IE, Suki D, Chang EL, Hassenbusch SJ, Weinberg JS, Shiu A, Maor MH, Woo SY (2005). Case-control study of stereotactic radiosurgery for recurrent Glioblastoma Multiforme. J Neurosurg.

[30] Ekici K, Ozseker N, Mayadagli A, Erdogan Kocak M, Olmezoglu A (2014). Efficacy of stereotactic radiotherapy as salvage treatment for recurrent malignant gliomas. J buon.

[31] Elliott RE, Parker EC, Rush SC, Kalhorn SP, Moshel YA, Narayana A, Donahue B, Golfinos JG (2011). Efficacy of gamma knife radiosurgery for small-volume recurrent malignant gliomas after initial radical resection. World Neurosurg.

[32] Niranjan A, Kano H, Iyer A, Kondziolka D, Flickinger JC, Lunsford LD (2015). Role of adjuvant or salvage radiosurgery in the management of unresected residual or Progressive Glioblastoma Multiforme in the pre-bevacizumab era. J Neurosurg.

[33] Frosina G. Radiotherapy of High-Grade gliomas: first half of 2021 update with special reference to Radiosensitization studies. Int J Mol Sci 2021, 22(16). 10.3390/ijms22168942 PMC8396323 34445646

[34] Cheon YJ, Jung TY, Jung S, Kim IY, Moon KS, Lim SH (2018). Efficacy of Gamma Knife Radiosurgery for Recurrent High-Grade Gliomas with Limited Tumor volume. J Korean Neurosurg Soc.

[35] Park KJ, Kano H, Iyer A, Liu X, Niranjan A, Flickinger JC, Lieberman FS, Lunsford LD, Kondziolka D (2012). Salvage gamma knife stereotactic radiosurgery followed by bevacizumab for recurrent Glioblastoma Multiforme: a case-control study. J Neurooncol.

[36] Guan Y, Xiong J, Pan M, Shi W, Li J, Zhu H, Gong X, Li C, Mei G, Liu X (2021). Safety and efficacy of hypofractionated stereotactic radiosurgery for high-grade gliomas at first recurrence: a single-center experience. BMC Cancer.

[37] Imber BS, Kanungo I, Braunstein S, Barani IJ, Fogh SE, Nakamura JL, Berger MS, Chang EF, Molinaro AM, Cabrera JR (2017). Indications and efficacy of Gamma Knife Stereotactic Radiosurgery for recurrent glioblastoma: 2 decades of institutional experience. Neurosurgery.

